# Supplementary material for: Roles of a Y-Linked iDmrt1 Paralogue and Insulin-like Androgenic Gland Hormone in Sexual Development in the Tropical Rock Lobster, Panulirus ornatus
Source: Int J Mol Sci. 2025 May 27;26(11):5149. doi: 10.3390/ijms26115149 (PMC12154352; doi:10.3390/ijms26115149)
Supplement: Supplementary file 1 [file ijms-26-05149-s001.zip › ijms-3554343-supplementary.pdf]

Supplementary Data S1

Searching *Po-iDMY* against the genome published by Ren *at al*, 2024

Sequences producing significant alignments Download Manage columns Show 100

select all 13 sequences selected GenBank Graphics

	Description	Max Score	Total Score	Query Cover	E value	Per. Ident	Acc. Len	Accession
☑	Panulirus ornatus isolate Po-2019 CTG_1661_pilon, whole genome shotgun sequence	171	171	43%	2e-47	100.00%	29101	JAZBNH010001417.1
☑	Panulirus ornatus isolate Po-2019 chromosome 50, whole genome shotgun sequence	135	271	60%	4e-35	77.01%	32424604	CM071302.1
☑	Panulirus ornatus isolate Po-2019 chromosome 50, whole genome shotgun sequence	135	271	60%	4e-35	77.01%	32424604	JAZBNH010000050.1
☑	Panulirus ornatus isolate Po-2019 chromosome 36, whole genome shotgun sequence	116	284	70%	2e-28	75.71%	19445244	CM071288.1
☑	Panulirus ornatus isolate Po-2019 chromosome 36, whole genome shotgun sequence	116	284	70%	2e-28	75.71%	19445244	JAZBNH010000036.1
☑	Panulirus ornatus isolate Po-2019 chromosome 64, whole genome shotgun sequence	54.3	54.3	19%	8e-07	65.79%	28623056	CM071316.1

Searching *Po-IAG* against the genome published by Ren *at al*, 2024

Sequences producing significant alignments Download Manage columns Show 100

select all 2 sequences selected GenBank Graphics

	Description	Max Score	Total Score	Query Cover	E value	Per. Ident	Acc. Len	Accession
☑	Panulirus ornatus isolate Po-2019 chromosome 38, whole genome shotgun sequence	112	276	79%	3e-27	100.00%	29624730	CM071290.1
☑	Panulirus ornatus isolate Po-2019 chromosome 38, whole genome shotgun sequence	112	276	79%	3e-27	100.00%	29624730	JAZBNH010000038.1

Supplementary Data S2

Repetitive elements distribution found using the automated Earl Grey annotation pipeline.

Classification	Total sequence length (bp)	Sequences count	Proportion of genome	Number of Distinct Classifications
DNA	66,868,503	112,253	2.72%	4,913
LINEs (Long interspersed nuclear elements)	131,002,933	221,365	5.33%	4,900
LTRs (Long terminal repeats)	21,321,817	30,897	0.87%	3,635
Other (Simple Repeat, Microsatellite, RNA)	2,923,528	1,579	0.12%	789
Penelope	30,995,988	75,236	1.26%	3,463
Rolling Circle	4,341,896	13,153	0.18%	1,736
SINEs (Short interspersed nuclear elements)	2,932,089	2,645	0.12%	1,193
Unclassified	395,807,973	480,390	16.11%	6,379

Supplementary Data S3

Chromium 10X genomic libraries (Illumina) data (1,473,906,560 read pairs), Pacific BioSciences genomic DNA library (PacBio) and RNA-Seq data from Ventura *et al*. (2020)[1] used in this study.

Library	Reads	Bases	Tissues
PacBio data (PRJNA952321)	7,620,300	44,015,913,196	Tail muscle (Adult male)
10X data (PRJNA952321)	1,473,906,560	221,085,984,000	Tail muscle (Adult male)
SRR22351161	39,452,638	5,917,866,333	Testis (Juvenile male)
SRR22351162	26,238,402	3,935,740,276	Testis (Juvenile male)
SRR22351164	58,855,224	8,828,240,776	Antennal gland (Adult male)
SRR22351165	33,717,902	5,057,652,760	Hemocytes (Adult male)
SRR22351168	25,351,644	3,802,722,198	Fat (Adult male)
SRR22351170	40,818,806	6,122,791,185	Epidermis (Adult male)
SRR22351172	49,675,366	7,451,256,687	Tail muscle (Adult male)
SRR22351174	55,765,796	8,364,829,173	Intestine (Adult male)
SRR22351176	31,760,590	4,764,057,709	Stomach (Adult male)
SRR22351178	31,582,816	4,737,391,931	Thoracic ganglia (Adult male)
SRR22351179	63,568,978	9,535,300,073	Heart (Adult male)
SRR22351182	21,251,654	3,187,727,227	Posterior gill (Adult male)
SRR22351183	65,759,046	9,863,808,728	Anterior gill (Adult male)
SRR22351191	23,673,258	3,550,965,898	Hepatopancreas (Adult male)
SRR22351192	33,278,740	4,991,786,626	Hepatopancreas (Adult male)
SRR22351193	44,547,374	6,682,062,706	Hepatopancreas (Adult male)
SRR22351194	53,099,808	7,964,932,305	Hepatopancreas (Juvenile male)
SRR22351195	37,836,394	5,675,431,500	Hepatopancreas (Juvenile male)
SRR22351200	36,264,894	5,439,699,166	Brain (Adult male)
SRR22351204	49,770,590	7,465,552,444	Distal spermduct (Adult male)
SRR22351205	42,330,738	6,349,579,831	Medial spermduct (Adult male)
SRR22351206	68,125,956	10,218,843,353	Proximal spermduct (Adult male)
SRR22351207	37,839,062	5,675,831,483	Testis (Adult male)
SRR22351208	23,201,602	3,480,223,382	Testis (Adult male)
SRR22351209	49,054,650	7,358,161,851	Testis (Adult male)
SRR22351210	26,209,550	3,931,413,151	Testis (Juvenile male)
SRR22351212	47,467,846	7,120,141,643	Eyestalk (Adult male)
SRR23142764	63,403,152	9,510,392,125	Antennules (Adult male)

Supplementary Data S4

> Po-iDMY Protein sequence from RACE 3’frame:
RHQQRLWRHLKDAKGRGDAIAEQSLADTKLQKCDMCRNHGVMKEKRAHKNTCPY
QDCPCDLCNLTRKRRDIMRHQQRVRRSQVTSRQHDEAYDYVIKTTAELAQMSMGT
TPAFNAPTPTSSFSRETATTDTTTTTNTTTTNTTTTITTATTTTITTATTITTTTTNSTCN
SASVPTECTSQTLASNTAMSVAPLKEPPPLVGNGLQF

Blast this protein sequence into the protein-coding of the male genome (Panulirus.ornatus_male.protein.fa), and find the protein sequence at the 5’frame as pictures below:

```
 Score = 39.7 bits (91), Expect = 0.17
 Identities = 19/35 (54%), Positives = 23/35 (65%)
 Frame = +2

Query: 1     MMNDDLTPGTRPAKPSGECVAYVYQDYELCVADDA 35
             MMNDDLTPGTRPAKPS + V         C+ +D+
Sbjct: 12941 MMNDDLTPGTRPAKPSKDKVDPSLSSSGSCMINDS 13045
```

Sbjct sequences

```
>scaffold_2464
          Length = 48196

 Score =  254 bits (649), Expect = 3e-66
 Identities = 128/128 (100%), Positives = 128/128 (100%)
 Frame = +1

Query: 166   RSQVTSRQHDEAYDYVIKTTAELAQMSMGTTPAFNAPTPTSSFSRETATTDTTTTTNTTT 225
             RSQVTSRQHDEAYDYVIKTTAELAQMSMGTTPAFNAPTPTSSFSRETATTDTTTTTNTTT
Sbjct: 46672 RSQVTSRQHDEAYDYVIKTTAELAQMSMGTTPAFNAPTPTSSFSRETATTDTTTTTNTTT 46851

Query: 226   TNTTTTITTATTTTITTATTITTTTTNSTCNSASVPTECTSQTLASNTAMSVAPLKEPPP 285
             TNTTTTITTATTTTITTATTITTTTTNSTCNSASVPTECTSQTLASNTAMSVAPLKEPPP
Sbjct: 46852 TNTTTTITTATTTTITTATTITTTTTNSTCNSASVPTECTSQTLASNTAMSVAPLKEPPP 47031

Query: 286   LVGNGLQF 293
             LVGNGLQF
Sbjct: 47032 LVGNGLQF 47055
```

Sbjct sequences

```
 Score =  201 bits (512), Expect = 3e-50
 Identities = 96/114 (84%), Positives = 98/114 (85%), Gaps = 13/114 (11%)
 Frame = +2

Query: 17    GECVAYVYQDYELCVADDAGQEGARNNKRQQHCTTCKNHGQNLRKSTHKCQYETCECLLC 76
             GECVAYVYQDYELCVADDAGQEGARNNKRQQHCTTCKNHGQNLRKSTHKCQYETCECLLC
Sbjct: 17249 GECVAYVYQDYELCVADDAGQEGARNNKRQQHCTTCKNHGQNLRKSTHKCQYETCECLLC 17428

Query: 77    QLTRLSRLVMRHQQRLWRHLKDAKGRGD-------------AIAEQSLADTKLQ 117
             QLTRLSRLVMRHQQRLWRHLKDAKGRGD             A+ EQ L D+KLQ
Sbjct: 17429 QLTRLSRLVMRHQQRLWRHLKDAKGRGDASRVYAAVGAAVAAMTEQGLTDSKLQ 17590
```

Sbjct sequences

```
 Score =  113 bits (282), Expect = 1e-23
 Identities = 48/50 (96%), Positives = 49/50 (98%)
 Frame = +2

Query: 117   QKCDMCRNHGVMKEKRAHKNTCPYQDCPCDLCNLTRKRRDIMRHQQRVRR 166
             QKCDMCRNHG+MKEKRAHKNTCPYQDC CDLCNLTRKRRDIMRHQQRVRR
Sbjct: 35744 QKCDMCRNHGIMKEKRAHKNTCPYQDCACDLCNLTRKRRDIMRHQQRVRR 35893
```

Sbjct sequences

> Complete Po-iDMY protein sequence

MMNDDLTPGTRPAKPSKDKVDPSLSSSGSCMINDSGECVAYVYQDYELCVADDAG
QEGERNNKRQQHCTTCKNHGQNLRKSNHKCQYEACECLLCQLTRLSRLVMRHQQR
LWRHLKDAKGRGDAIAEQSLADTKLQKCDMCRNHGVMKEKRAHKNTCPYQDCPC
DLCNLTRKRRDIMRHQQRVRRSQVTSRQHDEAYDYVIKTTAELAQMSMGTTPAFN

APTPTSSFSRETATTDTTTTTNTTTTNTTTTITTATTTTITTATTITTTTTNSTCNSASVP
TECTSQTLASNTAMSVAPLKEPPPLVGNGLQF

Blast this complete protein sequence into the male genome (Panulirus.ornatus_male.genomic.fna), getting the complete *Po-iDMY* sequence (ORF marked in red). Specific primers for sex markers were found at the C' tail of *Po-iDMY* (sequence marked in green).

ATGATGAATGATGACTTAACGCCAGGGACCAGACCTGCCAAGCCAAGTGGAGAG
TGTGTTGCTTATGTATATCAAGATTACGAACTTTGTGTTGCAGATGATGCAGGGC
AAGAGGGAGAGAGGAACAACAAGCGCCAACAACACTGCACCACATGCAAGAAC
CACGGGCAGAACTTGCGCAAGTCGAACCACAAGTGCCAGTACGAGGCCTGTGAG
TGCCTCTTGTGCCAGCTGACACGTCTCAGTCGCCTGGTGATGCGCCACCAACAGC
GCCTCTGGCGACACCTGAAGGACGCCAAGGGCCGGGGCGACGCCATCGCTGAAC
AGAGCCTTGCGGACACCAAGCTACAGAAGTGCGACATGTGCAGGAACCACGGCG
TTATGAAAGAGAAGCGGGCCCACAAGAACACCTGTCCCTACCAGGATTGTCCCT
GTGATCTGTGCAACCTCACCAGGAAGCGCCGGGACATAATGAGGCATCAGCAGC
GGGTCAGGAGGTCGCAAGTGACGAGCCGGCAACATGACGAGGCTTACGACTACG
TGATAAAGACCACGGCCGAATTGGCCCAGATGTCGATGGGCACCACTCCTGCCTT
TAACGCACCTACTCCAACCTCTAGTTTCTCCCGAGAAACCGCTACAACCGATACA
ACCACCACCACCAACACCACCACCACCAACACCACCACCACCATCACCACCGCC
ACCACCACCACCATCACCACCGCCACCACCATCACCACTACCACCAC**AAACTCG**
ACGTGCAACAGCGCCTCCGTCCCGACGGAGTGCACGTCGCAGACCCTGGCT
TCCAACACAGCCATGAGTGTCGCACCTCTCAAAGAACCCCCACCACTGGTTG
GTAACGGTTTACAATTCTAATATTAATATAACTAGAATTTGCTGCTAATGAC
GGTATGTCTGTGTTTCACTGTTAATATCTATCGTTCTTCATCTATACGTAATC
AGTTGTATATGAAATTACTTTTTTATCCCATTCAGGTGTATTTATACAACACT
GGGTACTTCCCAACCTTGGGCAAAGCCTTGACCCTTTCAAACTTCTTGAGAA
TGAAGATACGATCTGTGACCTTTGACCTGGTCCCTAAAGGGTCAGATGACA
GGCTATCACCATACCCTATCAATAAACACCTGAGACAATA

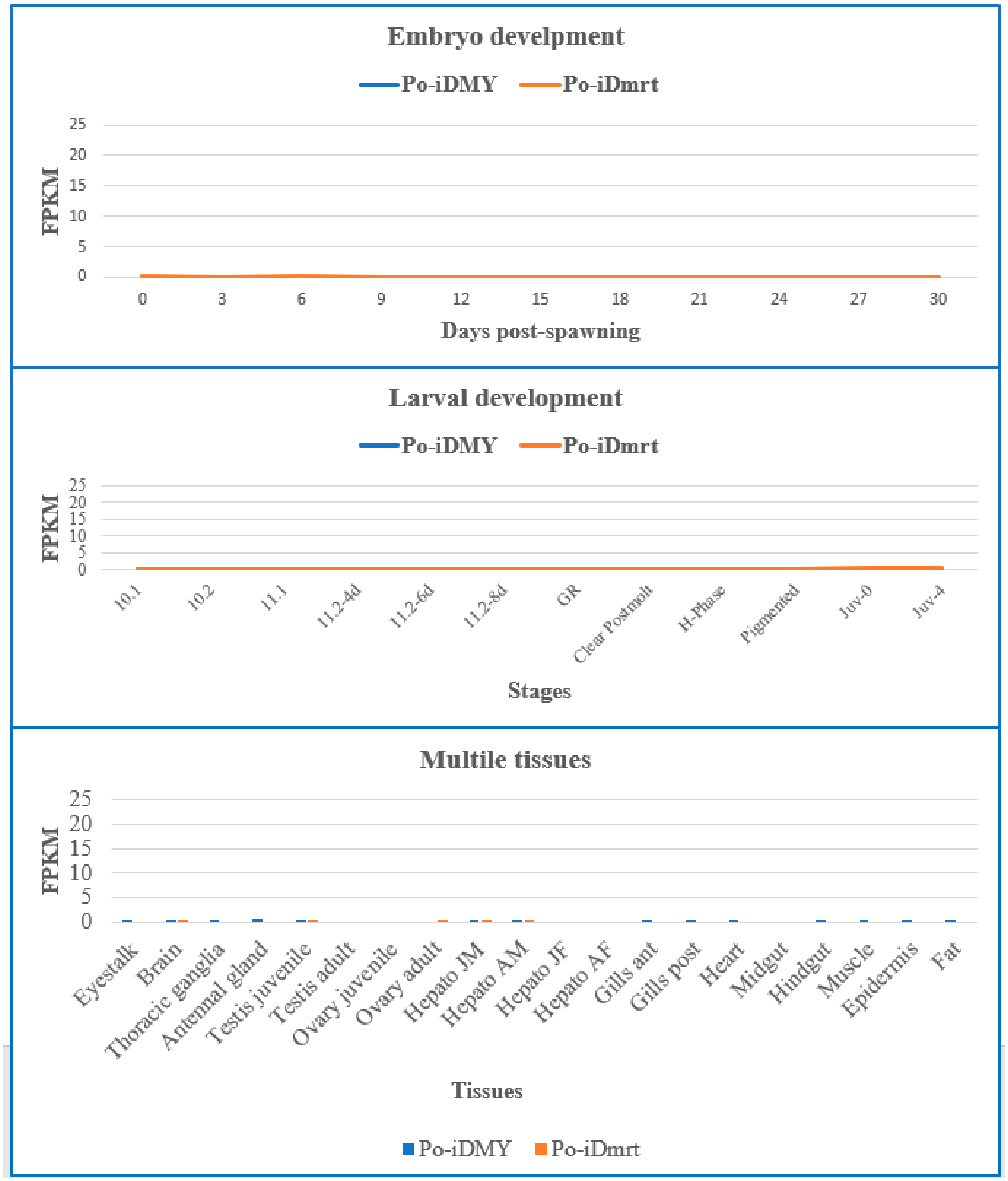

Figure S1. **The different expression patterns of *Panulirus ornatus Po-iDMY* and *Po-iDmrt* in embryos (A)[2], metamorphic stages (B)[3], multiple tissues (C)[1]**. FPKM – Fragments Per Kilobase of transcript per Million mapped reads; 10.1 – Phyllosoma larva stage 10, first instar; 10.2 – Phyllosoma larva stage 10, second instar; 11.1 – Phyllosoma larva stage 11, first instar; 11.2-4d – Phyllosoma larva stage 11, second instar, 4d post-molt; 11.2-6d – Phyllosoma larva stage 11, second instar, 6d post-molt; 11.2-8d – Phyllosoma larva stage 11, second instar, 8d post-molt; GR – Gut-retracting phyllosoma larva , the onset of metamorphosis; CP – Puerulus larva, 24h post-metamorphosis; H-Phase – Puerulus larva, 1-week post-metamorphosis; Pigmented – Puerulus larva, 2 weeks post-metamorphosis and <2 days pre-metamorphosis; Juv-0 – Juvenile lobster, 0-day post-metamorphosis; Juv-4 – Juvenile lobster, 4 days post-metamorphosis; Hepato JM – Hepatopancrease juvenile males;

Hepato AM – Hepatopancrease adult males; Hepato JF – Hepatopancrease juvenile females; Hepato AF – Hepatopancrease adult females.

Reference

1. Ventura T, Chandler JC, Nguyen TV, et al. Multi-Tissue transcriptome analysis identifies key sexual development-related genes of the ornate spiny lobster (*Panulirus ornatus*). *Genes*. 2020;11(10):1150.
2. Lewis CL, Fitzgibbon QP, Smith GG, Elizur A, Ventura T. Transcriptomic analysis and time to hatch visual prediction of embryo development in the ornate spiny lobster (*Panulirus ornatus)*. *Frontiers in Marine Science*. 2022:1009.
3. Hyde CJ, Fitzgibbon QP, Elizur A, Smith GG, Ventura T. Transcriptional profiling of spiny lobster metamorphosis reveals three new additions to the nuclear receptor superfamily. *BMC genomics*. 2019;20:531.

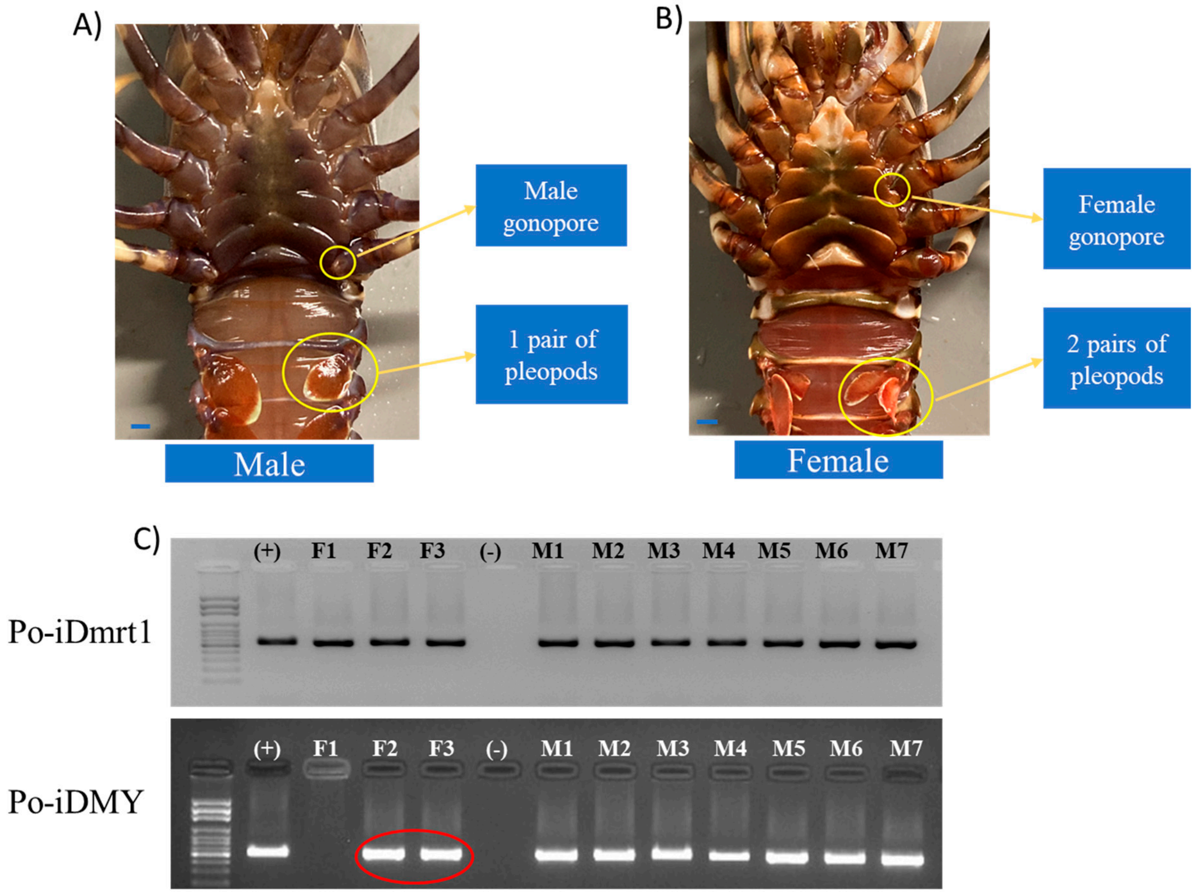

Figure S2. PCR of genomic DNA extracted from 10 Panulirus ornatus adults which were injected with dsRNA weekly to silence *IAG* gene for 2 months (from J1 to J4). This test was the second time, with the same result. M – Males; F – Females; (+): positive control (male sample); (-): negative control (water). Scale bar: 1mm.

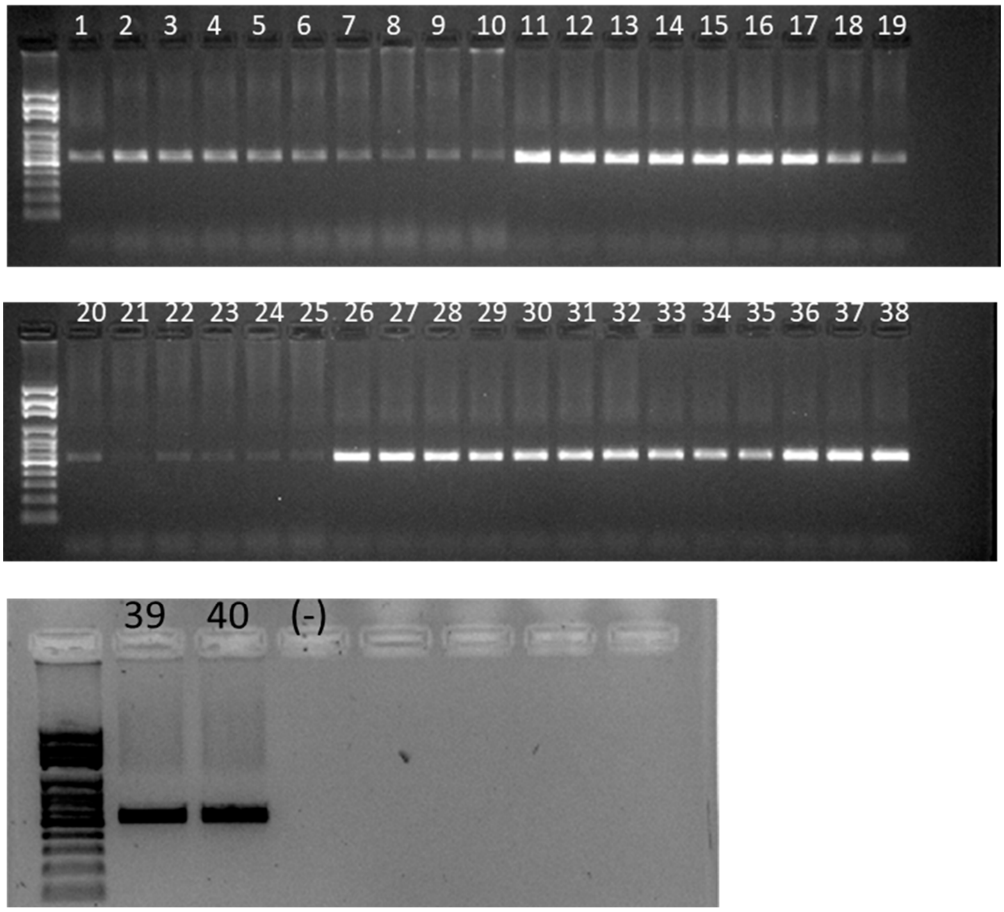

Figure S3. *Po-DMRT* was used as a positive control for female samples to ensure DNA integrity. All samples except sample 21 had a band at 600 bp for the positive control. However, this sample was successfully used to amplify the Po-iDMY gene (See Figure 2) indicating the intact DNA of this sample. Number (1,2…40) – sample order: (-) – negative control.
